# Comparative Analysis of Physicochemical Properties, Antioxidant and Enzyme Inhibitory Activities of Sporoderm-Broken *Ganoderma lucidum* Spore Powders from Different Regions in China

**DOI:** 10.3390/foods15091579

**Published:** 2026-05-04

**Authors:** Jingxiao Li, Ru Li, Huabin Zhou, Hang Qu, Bo Chen, Hailong Yang

**Affiliations:** 1 College of Life & Environmental Science, Wenzhou University, Wenzhou 325035, China; ljx828828@163.com (J.L.); 18536474845@163.com (R.L.); zhb@wzu.edu.cn (H.Z.); 20210032@wzu.edu.cn (H.Q.); 2 Zhejiang Provincial Key Laboratory for Water Environment and Marine Biological Resources Protection, Wenzhou University, Wenzhou 325035, China; 3 Zhejiang Yiming Food Co., Ltd., Wenzhou 325000, China

**Keywords:** *Ganoderma lucidum* spore powder, different region, triterpenoids, polysaccharides, bioactivity

## Abstract

*Ganoderma lucidum* spore powder is widely recognized as a high-grade *Ganoderma* product and is extensively consumed as a functional food and dietary supplement in China. To compare quality differences, nine batches of sporoderm-broken *G. lucidum* spore powders (DX, SD, FJ, JL, XZ, LQ, AH, LN, and GZ) were collected from representative producing regions across China. Their physicochemical properties, antioxidant activities, and enzyme inhibition capacities were analyzed in this work. The results revealed varying degrees of differences in color, chemical composition, antioxidant activity, and metabolic enzyme inhibitory effects among the samples. Notably, sample GZ contained the highest levels of total sugar, polysaccharides, lipids, protein, total phenolics, and total triterpenoids; sample XZ had the highest ergosterol content; and sample LN exhibited the highest levels of reducing sugar and nucleosides. GZ demonstrated the strongest radical scavenging activity, ferric-reducing antioxidant power (FRAP), cupric ion-reducing capacity, and inhibitory effects against α-glucosidase, α-amylase, lipase, acetylcholinesterase, and xanthine oxidase. Sample AH showed the greatest Fe^2+^-chelating capacity. Principal component analysis indicated that GZ, AH, and LN exhibited stronger antioxidant and metabolic enzyme inhibition activities, whereas LQ and FJ showed lower activities. These findings confirm significant quality differences among *G. lucidum* spore powders sourced from different geographical regions.

## 1. Introduction

*Ganoderma lucidum*, one of the most renowned edible and medicinal fungi, has been used as traditional Chinese medicine and functional food in China, South Korea, and Japan for thousands of years [1]. *G. lucidum* spores are minute, ovoid germ cells released from mature *G. lucidum* fruiting bodies and are widely regarded as the bioactive essence [2]. Accordingly, the chemical components and biological activities of *G. lucidum* spores have been extensively investigated. Numerous studies have confirmed that *G. lucidum* spores harbor a variety of bioactive compounds and exhibit multiple pharmacological effects, including antitumor, immunomodulatory, anti-inflammatory, antioxidant, neuroprotective, hepatoprotective, hypoglycemic, and hypolipidemic activities [2,3,4]. Notably, *G. lucidum* spores contain some unique compounds (e.g., lucidenic acid SP1, ganosporelactone A, and ganosporelactone B) that are not present in *G. lucidum* fruiting bodies [4], endowing them with superior bioactivity and medicinal value compared to the corresponding fruiting bodies [5].

As an important Chinese medicine material and functional food ingredient, *G. lucidum* is widely cultivated in China. According to statistics from the Chinese Edible Fungi Association (https://bigdata.cefa.org.cn/output.html, accessed on 7 March 2026), the annual production of *G. lucidum* in China has exceeded 200,000 tons since 2021. *G. lucidum* spores are produced as a premium product, with cultivation areas spanning more than 20 provinces, including Shandong, Zhejiang, Heilongjiang, Guizhou, and Anhui provinces, and the Xizang Tibetan autonomous region. Notably, over 60% of China’s total *G*. *lucidum* spore powder output is concentrated in four major producing regions: Wuyi Mountain (Fujian Province) and Longquan region (southwest Zhejiang Province), Changbai Mountain of northeast China, Dabie Mountain of Anhui Province, and the western region of Shandong Province [6]. Currently, *G. lucidum* spore products are extremely popular in the Chinese medicine and healthcare markets, with an annual output value exceeding USD 1.3 billion [7]. Market demand continues to grow steadily due to their well-documented health-promoting effects [8].

It is well established that geographical origin profoundly influences the phytochemical profiles and bioactive potency of medicinal and edible plant materials [9]. For instance, Tamma et al. compared the chemical composition and biological properties of *Thymus capitatus* collected from different regions of Algeria and reported that the sample from Guelma region exhibited the highest flavonoid content and antioxidant activity [10]. Similarly, research by Wang et al. demonstrated that Chinese water chestnuts cultivated in Hefei (Anhui Province, China) contained higher phenolic content and exhibited stronger radical scavenging activity than those from other producing regions in China [11]. Li et al. Further showed that *Radix puerariae* sourced from Guangzhou had the highest phenolic content and the strongest inhibitory activity against carbohydrate-hydrolyzing enzymes among seven samples collected across China [12]. Comparable effects of geographical conditions on phenolic profiles and antioxidant capacity have also been reported for *Citrus medica* from China [13], *Tanacetum parthenium* from central Ukraine [14], and *Codonopsis lanceolata* from South Korea [15]. The producing regions of *G. lucidum* spores span most provincial-level areas of China, encompassing highly diverse geographical and climatic conditions. Key ecological factors for *G. lucidum* cultivation, including altitude, temperature, light, water availability, and nutrient status, vary substantially across these regions. Additionally, *G. lucidum* strains cultivated in different areas may possess distinct genetic characteristics, and cultivation management and processing techniques also differ widely among producing regions. Therefore, we hypothesized that the physicochemical and bioactive properties of *G. lucidum* spores from different geographical regions of China would differ significantly.

In this study, we analyzed sporoderm-broken *G. lucidum* spore powders collected from nine representative cultivation regions across China. The primary objective was to systematically compare the regional differences in appearance, chemical composition, antioxidant activity, and inhibitory effects against five metabolic enzymes: α-glucosidase, acetylcholinesterase, α-amylase, lipase, and xanthine oxidase. Our findings provide a scientific basis for the quality control and high-value functional application of *G. lucidum* spore powder.

## 2. Materials and Methods

### 2.1. G. lucidum Spore Powder Samples

**Figure 1:**
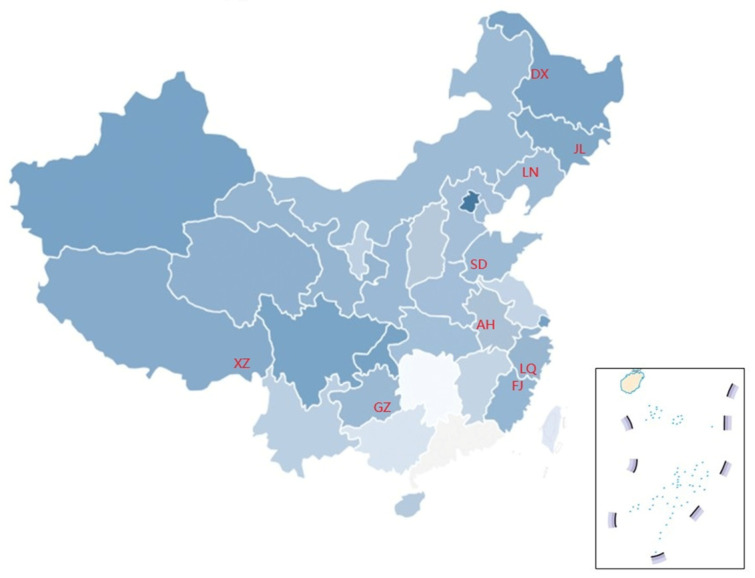
Map of China indicating the geographic regions of *G. lucidum* spore powder samples. Note: JL, AH, SD, DX, GZ, LN, FJ, XZ, and LQ represent samples collected from the provinces of Jilin, Anhui, Shandong, Heilongjiang, Guizhou, Liaoning, Fujian, Xizang, and Zhejiang, respectively.

Nine sporoderm-broken *G. lucidum* spore powder samples were collected from representative cultivation regions across China in November 2024. The sampling sites included: Daxinganling (Heilongjiang Province, Northeast China), Changbai mountain (Jilin Province, Northeast China), Shenyang (Liaoning Province, Northeast China), Guan county (Shandong Province, East China), Wuyi mountain (Fujian Province, Southeast Chins), Longquan (Zhejiang Province, Southeast China), Nyingchi Prefecture (Xizang Tibetan Autonomous Region, West China); Dabie mountain (Anhui Province, Central China), and Qiannan Miao and Buyi autonomous prefecture (Guizhou Province, Southwest China). The nine samples were designated as DX, JL, LN, SD, FJ, LQ, XZ, AH, and GZ, respectively, and their corresponding geographical locations are shown in Figure 1.

### 2.2. Sporoderm-Broken Rate Determination and Color Analysis

Each sample (5.0 g) was dried at 65 °C for 48 h. 5.0 mg aliquot of spore powder was fixed on aluminum sample table, sputter-coated with gold under vacuum, and then observed using a Regulus 8100 scanning electron microscope (Hitachi, Tokyo, Japan). The acceleration voltage was set to 3.0 kV, and the observation magnification was 800×. The sporoderm-broken rate of each sample was calculated by counting and evaluating 100 randomly selected spores per sample.

The color values (L*, a*, and b*) of each sample were measured via reflection mode using a CR-5 colorimeter (Konica Minolta, Tokyo, Japan), with 10 replicate measures performed for each sample.

### 2.3. Determination of Total Sugar, Reducing Sugar, and Polysaccharide Contents

A 2.0 g aliquot of each sample was subjected to reflux extraction twice with 40 mL of deionized water at 85 °C for 4 h per cycle. After filtration, the filtrates from the two extraction cycles were combined and concentrated, and the resulting solution was used directly for the measurement of total sugar and reducing sugar contents. For polysaccharide content determination, 40 mL of the combined filtrate was concentrated to 10 mL under vacuum and 50 °C. The polysaccharides were then precipitated by adding 40 mL of anhydrous ethanol, washed with 80% (*v*/*v*) ethanol, and finally redissolved in deionized water for subsequent analysis.

The phenol-sulfuric acid method was used to determine total sugar and polysaccharide contents, while the DNS method was employed for reducing sugar content determination [16].

### 2.4. Determination of Total Triterpenoid, Total Phenolic, and Individual Phenolics Contents

A 1.0 g aliquot of each sample was ultrasonically extracted with 20 mL of 80% (*v*/*v*) ethanol at 40 °C for 60 min, with an ultrasonic power of 480 W and frequency of 40 kHz. The supernatant was collected by centrifugation and used for the following determination.

The triterpenoid content was determined using the vanillin-acetic acid-perchloric acid method as describe by Yan et al. [17]. The total phenolic content was measured using the Folin–Ciocalteu method [1]. Individual phenolic compounds were analyzed via HPLC as described by Dong et al. [1]. Hydroxybenzoic acid, gallic acid, vanillic acid, kaempferol, and protocatechuic acid were quantified using the external standard method, respectively.

### 2.5. Determination of Protein and Lipid Contents

A 0.5 g aliquot of each sample was digested with copper sulfate, potassium sulfate, and concentrated sulfuric acid. The protein content was then determined using acetylacetone formaldehyde spectrophotometry in accordance with the Chinese National Food Safety Standard GB 5009.5-2016 [18].

A 2.0 g aliquot of each sample was extracted with 150 mL of petroleum ether using a Soxhlet system. After extraction, the solvent was removed via evaporation using a rotary evaporator (RE-2000, Shanghai Yarong Biochemical Instrument Co., Shanghai, China), and the lipid content was determined via the gravimetric method.

### 2.6. Determination of Ergosterol Content

A 10 g aliquot of each sample was ultrasonically extracted with 300 mL of petroleum ether for 30 min. The supernatant was collected by centrifugation, concentrated to dryness under reduced pressure at 30 °C, and redissolved in a chloroform-methanol mixture (1:1, *v*/*v*). The ergosterol content was determined by an Agilent 1260 Infinity II HPLC (Agilent Technologies, Santa Clara, CA, USA), and quantified via external standard method as describe by Ran et al. [19].

### 2.7. Determination of Nucleoside Contents

A 2.0 g aliquot of each sample was extracted by ultrasonication with 15 mL of ultrapure water for 1 h. The supernatant was collected by centrifugation for HPLC analysis. Nucleoside separation and quantification were performed using gradient elution on a ZORBAX Eclipse Plus C18 column (4.6 × 250 mm, 5 μm), with a modified method based on Khan et al. [20]. Briefly, the mobile phase consisted of ultrapure water (A) and methanol (B) at a flow rate of 1 mL/min. The gradient elution program was set as follows: 0–10 min, 0% B; 10–15 min, 0% B to 5% B; 15–30 min, 5% B to 20% B; 30–31 min, 20% B to 0% B; 31–35 min, 0% B. The injection volume, column temperature, and detection wavelength were 10 μL, 30 °C, and 259 nm, respectively. Nucleoside contents were calculated using the external standard method.

### 2.8. Determination of Mineral Elements

A 0.5 g aliquot of each sample was mixed with 5 mL of nitric acid, and subjected to microwave digestion for 25 min at 800 W using a MARS 6 microwave digestion system (CEM Corporation, Matthews, NC, USA). The excess acid was then evaporated to dryness at 150 °C. After cooling, the residue was redissolved in ultrapure water. Elemental analysis was conducted using an inductively coupled plasma mass spectrometer (iCAP™ TQ ICP-MS, Thermo Fisher Scientific, Waltham, MA, USA) under the following operating conditions: RF power 1550 W, nebulizer gas flow 1.0 L/min, auxiliary gas 0.8 L/min, plasma gas flow 14 L/min, nebulizer pump speed, 40 r/min, and analysis mode kinetic energy discrimination (KED). The contents of 15 target mineral elements were quantified individually via the external standard method.

### 2.9. Determination of Antioxidant Activity

The free radical scavenging activities of 2,2-diphenyl-1-picrylhydrazyl (DPPH) and 2,2′-azino-bis (3-ethylbenzothiazoline-6-sulfonic acid) (ABTS), as well as the ferric-reducing antioxidant power (FRAP), were determined colorimetrically following the method described by Dong et al. [1]. Vitamin C was used as the reference antioxidant, and the results were expressed as mg of vitamin C equivalents per gram of sample (mg VCE/g).

The cupric ion-reducing antioxidant capacity (CUPRAC) and Fe^2+^-chelating ability were evaluated in 96-well microplates with reference to the method of Quradha et al. [21]. For the CUPRAC assay, 50 μL of 1 mol/L NH_4_CH_3_COOH buffer (pH 7), 50 μL of 10 mmol/LCuSO_4_ solution, 50 μL of 7.5 mmol/L neocuproine solution, and 100 μL of sample extract were mixed. The mixture was incubated for 30 min, and then absorbance was recorded at 450 nm using a Spectra MAX 190 microplate reader (Molecular Devices, San Jose, CA, USA). Vitamin C was used as the positive control, and the results were expressed as mg of vitamin C equivalents per gram of sample (mg VCE/g). For the ferrous ion-chelating assay, 150 μL of extract solution, 30 μL of 0.2 mg/mL FeCl_2_ solution, and 70 μL of 2 mg/mL ferrozine solution were sequentially mixed, incubated for 10 min, and absorbance was recorded at 560 nm using the same microplate reader. Results were expressed as mg of EDTA equivalents per gram of sample (mg EDTA/g).

### 2.10. Determination of Enzyme Inhibitory Activity

The sample extract for enzyme inhibition assays was prepared as described in Section 2.4. The extract was evaporated to dryness under vacuum, and redissolved in dimethyl sulfoxide (DMSO) for subsequent analysis [22].

The inhibitory activities against α-glucosidase and α-amylase were measured as described by Zhao et al. [16] and Maccarronello et al. [23], respectively. The results were expressed as mg of acarbose equivalents per gram of sample (mg ACE/g).

The lipase inhibitory activity was determined in 96-well microplates using a modified method based on Zhao et al. [16]. Briefly, 30 μL of 3500 U/mL pancreatic lipase solution, 60 μL of sample solution, and 80 μL of 0.1 mol/L PBS (pH 7.7) were mixed and incubated at 37 °C for 20 min. Subsequently, 40 μL of 0.8 mg/mL 4-nitrophenyl laurate solution was added to the mixture. After an additional 120 min incubation at 37 °C, the absorbance at 405 nm was measured. The results were expressed as μg of orlistat equivalents per gram of sample (μg ORE/g).

The acetylcholinesterase (AChE) inhibitory activity was determined by the method of Sansenya & Payaka [24], and results expressed as μg of galantamine equivalents per gram of sample (μg GTE/g). The xanthine oxidase inhibition activity was measured using the method of Ma et al. [25], with results expressed as μg of allopurinol equivalents per gram of sample (μg APE/g).

### 2.11. Statistical Analysis

All experiments were performed in triplicate, except for the sporoderm-broken rate determination and color analysis. Results were presented as mean ± standard deviation. Statistical significance was analyzed using GraphPad Prism 10.1.2 (GraphPad Software, Boston, MA, USA) via one-way analysis of variance (ANOVA) followed by Tukey’s Honest Significant Difference (HSD) test. Differences with *p* < 0.05 were considered as statistically significant. Principal component analysis (PCA) was performed using OriginPro 2021 (OriginLab Corporation, Northampton, MA, USA).

## 3. Results and Discussion

### 3.1. Sporoderm-Broken Rate and Color

*G. lucidum* spores are encapsulated by outer bilayers of sporoderm, and the sporoderm-broken rate directly determines the release efficiency of intracellular bioactive ingredients [2]. Sriket et al. reported that the total phenolic and flavonoid contents in *G. lucidum* G2 spores increased from 0.14 mg GAE/g and 0.17 mg QE/g to 4.11 mg GAE/g and 6.29 mg QE/g, respectively, after sporoderm breakage via vibratory milling [26]. Another study demonstrated that the bioaccessibility of polysaccharides and triterpenes from *G. lucidum* spores increased from 29.52% to 44.53–104.18% and from 5.38% to 12.96–32.90%, respectively, after low-temperature ultra-fine grinding to break the sporoderm [27]. As shown in Table 1, the sporoderm-broken rate of the nine samples ranged from 94.08% to 99.98%, with sample FJ and sample GZ exhibiting the lowest and the highest values, respectively. However, no statistically significant differences were observed among the nine samples (*p* > 0.05).

Generally, *G. lucidum* spores are brown or dark brown in appearance [4]. To accurately characterize the color differences among samples, the L* (lightness), a* (redness), and b* (yellowness) values of all samples were determined, with the results listed in Table 1. The L* values followed the order of GZ > FJ > LQ > AH > JL > SD > XZ > DX > LN. No significant differences in L* values were observed among GZ, FJ, and LQ (*p* > 0.05), nor among SD, XZ, and DX (*p* > 0.05). Sample FJ exhibited the highest a* and b* values, while sample LN showed the lowest values for both parameters. The ranking order of the samples for a* and b* values was nearly identical, with the only exception being sample LQ, which ranked 4th for a* value but 7th for b* value. These data indicated differences in color among the nine samples. The color of sporoderm-broken *G. lucidum* spore powder is mainly affected by multiple factors, including raw material origin, drying process, and breaking technology. Consistent with our findings, Zhang et al. [6] also observed significant differences in color values among nine sporoderm-broken *G. lucidum* spore powders collected from the Dabie Mountain, Wuyi Mountain, Changbai Mountain, and the western region of Shandong Province in China.

### 3.2. Total Sugar, Reducing Sugar, Protein, and Lipid Contents

Sugar, protein, and lipids are the primary nutritional components of *G. lucidum* spore powder. As shown in Table 2, the total sugar content of the nine samples ranged from 13.55 mg/g to 36.02 mg/g, in the following descending order: GZ > LN > JL > SD > XZ > AH > DX > LQ > FJ. The reducing sugar content ranged from 3.20 mg/g to 9.16 mg/g, with the sequence LN > GZ > AH > FJ > XZ > LQ > DX > SD > JL. Protein content ranged from 1.96% to 15.35%, in the order GZ > LN > LQ > AH > DX > JL > XZ > SD > FJ. Lipid content ranged from 30.65% to 37.94%, following the sequence GZ > SD > XZ > LN > DX > FJ > JL > AH > LQ. Among the nine samples, GZ exhibited the highest contents of total sugars, protein, and lipids, while LN had the highest reducing sugar content. Zhang et al. [6] determined the lipid content of nine *G. lucidum* spore powder samples from different producing regions in China and found that the sample from western Shandong Province had the highest lipid content, whereas the sample from Wuyi Mountain had the lowest. Consistent with our results, Gong et al. [28] also detected differences in total sugar, protein, and lipid contents among six *G. lucidum* spore powder samples collected from Shandong Province, China.

### 3.3. Polysaccharide, Triterpenoid, and Ergosterol Contents

Polysaccharides and triterpenoids are the core bioactive components of *G. lucidum* spore powder and have been demonstrated to exhibit a wide range of biological activities, including antitumor, immunomodulatory, anti-inflammatory, anti-aging, free-radical scavenging, and hypolipidemic effects [2,29,30,31]. As shown in Table 3, the polysaccharide content of the samples ranged from 7.28 mg/g to 27.47 mg/g, representing a 3.77-fold difference between the highest and lowest values among the nine samples, with the following order: GZ > LN > SD > AH > XZ > JL > DX > LQ > FJ. The total sugar content ranged from 13.55 mg/g to 36.02 mg/g, following the sequence: GZ > LN > JL > SD > XZ > AH > DX > LQ > FJ. The reducing sugar content ranged from 3.20 mg/g to 9.16 mg/g, with the order: LN > GZ > AH > FJ > XZ > LQ > DX > SD > JL. The highest triterpenoid content was found in sample GZ (14.73 mg/g), followed by LN > XZ > DX > AH > SD > JL > LQ, while sample FJ had the lowest triterpenoid content (7.73 mg/g). The bioactive component contents of *G. lucidum* spore powder vary depending on factors such as sample source, cultivation substrate, and drying process. Zhong et al. analyzed 59 *G. lucidum* spore powder samples from Zhejiang, Jilin, Jiangsu, Anhui, Shandong, Yunnan, and Hubei provinces in China and reported that the polysaccharide content ranged from undetectable to 7.20% [32]. The polysaccharide contents of six *G. lucidum* spore powder samples from Shandong Province were determined to be 0.88–1.18% [28]. Data from Zhang et al. showed that the polysaccharide content of five commercial *G. lucidum* spore powder products ranged from 1.05% to 1.87% [33]. Chang et al. [29] reported that the tree species used for cultivation and harvest time significantly affected the polysaccharide and triterpenoid contents in *G. lucidum* spore powder. Consistent with these previous reports, significant differences in polysaccharide and triterpenoid contents were observed among the nine samples in this study (*p* < 0.05), with the highest values detected in GZ (27.47 mg/g and 14.73 mg/g, respectively) and the lowest in FJ (7.28 mg/g and 7.73 mg/g, respectively) (Table 3).

Ergosterol is the predominant sterol in *G. lucidum* spore oil and has been reported to possess multiple biological activities, such as antitumor, antioxidant, hypoglycemic, and hypocholesterolemic effects [34,35]. A previous study found that the ergosterol content of *G. lucidum* spore powder from Nanjing (Jiangsu Province, China) ranged from 1.27 to 1.44 mg/g, varying with different extraction methods [34]. In this work, the ergosterol content of the nine samples ranged from 0.30 mg/g to 3.41 mg/g, in the following descending order: XZ > SD > GZ > JL > LN > LQ > FJ > AH > DX (Table 3). Statistically significant differences were detected among the samples (*p* < 0.05), with no significant difference observed among samples JL, LN, LQ, and FJ.

### 3.4. Total Phenolic, and Individual Phenolics Contents

The fruiting bodies and spores of *G. lucidum* contain a variety of phenolic compounds (including protocatechuic acid, hydroxybenzoic acid, coumaric acid, etc.), that are the primary contributors to its antioxidant activity [36,37]. In this work, the total phenolic content of the nine samples ranged from 106.69 μg/g to 351.07 μg/g, representing a 3.29-fold difference between the highest and lowest values. The order of phenolic content was GZ > AH > LN > JL > SD > DX > XZ > LQ > FJ (Table 3). These values were markedly higher than the total phenolic content (0.61 mg/100 g) reported in *G. lucidum* spores from Bragança, Northeast Portugal [36], which may be attributed to differences in sample origin, collection time, and analytical methods.

Five individual phenolic compounds, including protocatechuic acid, hydroxybenzoic acid, vanillic acid, kaempferol, and gallic acid, were quantified in this study, with the results presented in Table 4. Consistent with the findings of Taofiq et al. [38], protocatechuic acid and hydroxybenzoic acid were the main phenolic compounds in the samples. Protocatechuic acid was detected in all nine samples, with the highest content (50.87 μg/g) in sample GZ and the lowest (7.48 μg/g) in sample XZ. Hydroxybenzoic acid was detected in eight samples, with contents ranging from 3.67 μg/g to 9.97 μg/g, while it was not detected in sample XZ. Kaempferol was found in seven samples, with contents of 10.17–26.51 μg/g, and was absent in samples SD and LQ. Gallic acid was detected in six samples, ranging from 0.71 μg/g to 3.99 μg/g, and was not detected in samples AH, SD, and XZ. Vanillic acid was detected only in four samples: SD (7.05 μg/g), GZ (7.05 μg/g), LN (4.84 μg/g), and XZ (7.88 μg/g).

### 3.5. Nucleoside Components

Nucleosides represent another important class of bioactive compounds in *G. lucidum* spore powder, with documented antitumor, antiviral, and central nervous system sedative effects [39,40]. Wang et al. analyzed 15 nucleosides in *G. lucidum* spore powder and identified uridine, guanosine, and adenosine as the predominant nucleoside components [39]. In this study, six nucleosides were quantitatively analyzed. Uracil, adenosine, and uridine were detected in all nine samples, with contents ranging from 3.51 μg/g to 51.98 μg/g, 11.39 μg/g to 144.53 μg/g, and 23.65 μg/g to 210.41 μg/g, respectively. Guanosine was detected in eight samples (all except SD), with contents of 10.11–36.56 μg/g. Inosine was found in five samples (GZ, XZ, FJ, LQ, and LN), with contents ranging from 8.38 μg/g to 18.33 μg/g. Guanine was detected in six samples (AH, DX, JL, SD, XZ, and LN), with contents of 4.59–100.02 μg/g (Table 5). Collectively, these results indicated significant differences in nucleoside profiles and concentrations among *G. lucidum* spore powder samples from different geographical regions in China.

### 3.6. Mineral Elements

Mineral elements such as Ca, Mg, K, and Fe play vital roles in the growth and metabolism of *G. lucidum*. For instance, Cu is an essential element for the synthesis of laccase, a key enzyme involved in lignocellulose degradation by *G. lucidum* [41]. Additionally, *G. lucidum* can accumulate trace and/or potentially toxic elements from its cultivation environment, including the substrate, soil, and water, during its growth [42]. A previous study found that *G. lucidum* spore powders cultivated on different tree species and harvested at different times contained varying levels of Cr, Ni, As, Cd, and Pb [29]. In this work, 14 mineral elements in *G. lucidum* spore powder samples were analyzed, and the results are presented in Table 6. The contents of B, Mg, K, Ca, Fe, Se, Mn, Ba, Cu, As, Ti, Cd, Cr, and Pb ranged from 0.58 to 8.11, 9.47 to 417.61, 100.71 to 315.59, 15.86 to 648.89, 3.24 to 47.39, 0.07 to 0.50, 2.04 to 5.79, 0.94 to 8.42, 13.49 to 20.39, 0.05 to 0.20, 0.31 to 1.89, 0.05 to 0.23, 0.12 to 1.25, and 0.03 to 0.52 mg/kg, respectively. These values indicated fold differences of 13.98, 44.10, 3.13, 40.91, 14.63, 7.14, 2.84, 8.96, 1.51, 4.0, 6.10, 4.60, 10.42, and 17.33 among the nine samples collected from different geographical regions.

Among the samples, LQ contained the highest Ba content and the lowest levels of B, Mg, K, Ca, Mn, As, Ti, and Pb; AH had the lowest Cu content and the highest contents of K, Ca, Mn, Cd, and Cr; SD showed the highest Cu content and the lowest contents of Fe, As, and Cd; LN contained the highest contents of Mg, Se, Ti, and Pb; GZ had the highest contents of B and Fe; and FJ exhibited the highest As content. The lowest contents of Cr, Se, and Ba were detected in JL, DX, and XZ, respectively. The contents of Pb, Cd, Cu, Cr, As, and Se in *G. lucidum* spore powder reported by Mu et al. [18] were 0.197, 0.152, 16.166, 0.856, 0.199, and 0.050 mg/kg, respectively, all within the ranges detected in this study. Additionally, Pb, Cd, and As are recognized as potentially toxic elements [42]. The maximum contents of these elements in the samples were 0.52 mg/kg (Pb), 0.23 mg/kg (Cd), and 0.20 mg/kg (As), respectively. These values were substantially lower than those reported that by Xu and Li [4], who found 10.49 mg/kg for Pb, 0.65 mg/kg for Cd, and 0.34 mg/kg for As. Importantly, all detected levels of these potentially toxic elements did not exceed the maximum permissible limits established by the current applicable standards in China, including the Industry Standards for Supply and Marketing Cooperatives “*Ganoderma lucidum* wall-broken spores powder” (GH/T 1133-2017) [43], Local Standards in Guizhou Province “*Ganoderma lucidum*” (DBS52/057-2022) [44], Local Standards in Zhejiang Province “*Ganoderma lucidum*” (DBS33/3020-2025) [45], and Local Food Safety Standards of Shandong Province “*Ganoderma lucidum*” (DBS37/003-2024) [46].

### 3.7. Antioxidant Activity

**Figure 2:**
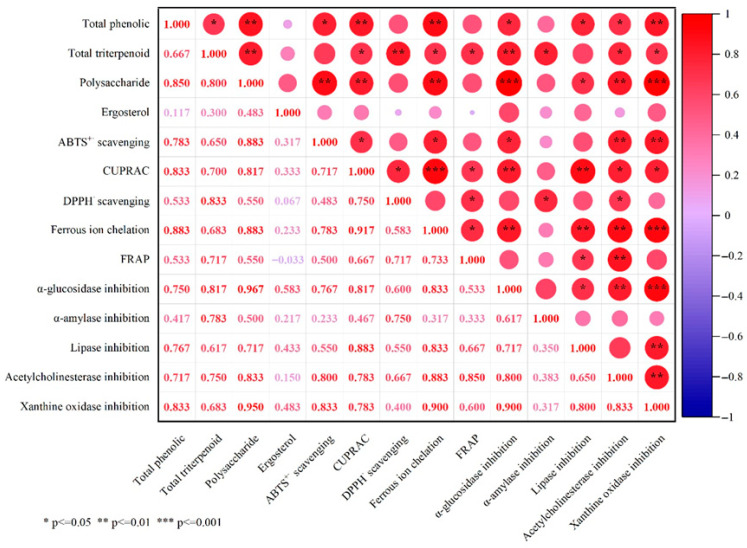
Correlation analysis of bioactive compounds and biological activities in *G. lucidum* spore powder samples collected from different regions of China.

Oxidative stress is closely associated with the onset and progression of a series of chronic degenerative diseases, and antioxidant activity is one of the core functional properties of *G. lucidum* spore powder [2,40]. Previous studies have confirmed significant differences in the antioxidant capacity of *G. lucidum* spore powders from different geographical origins [6]. In this study, the antioxidant capacity of the tested *G. lucidum* spore powder samples was evaluated using five complementary assays: DPPH· scavenging activity, ABTS^+^· radical scavenging activity, FRAP, CUPRAC, and Fe^2+^-chelating activity. As shown in Table 7, the nine samples exhibited distinct antioxidant capacities across the five assays. Among them, sample GZ showed the highest CUPRAC value (4.02 mg VCE/g), as well as the strongest DPPH· and ABTS^+^· scavenging activities (0.40 mg VCE/g and 1.46 mg VCE/g, respectively). These values were 2.59-, 1.54-, and 1.92-fold higher than the lowest values, which were detected in samples LQ, SD, and FJ, respectively. The highest FRAP value (0.42 mg VCE/g) was observed in both GZ and AH, which was 1.4-fold higher than the lowest value determined in sample JL. The strongest Fe^2+^-chelating activity (0.72 mg EDTA/g) was found in sample AH, which was 3.43-fold higher than the lowest value detected in sample LQ. Previous studies have indicated that the antioxidant activity of *G. lucidum* fruiting bodies and spores correlates with their contents of phenolics, triterpenoids, and polysaccharides [1,36], owing to the abundant reducing end groups and active hydroxyl groups presented in these compounds [47]. Consistently, the DPPH· scavenging activity, ABTS^+^· scavenging activity, CUPRAC, and Fe^2+^-chelating activity of the nine samples showed strong correlations with total triterpenoid content, polysaccharide content, total phenolics/polysaccharide contents, and total phenolics/polysaccharide contents, with correlation coefficients of 0.833, 0.883, 0.833/0.817, and 0.883/0.883, respectively (Figure 2).

### 3.8. Enzyme Inhibitory Activity

In recent decades, the global prevalence of chronic metabolic and degenerative diseases, including type II diabetes, obesity, Alzheimer’s disease, and hyperuricemia, has been steadily increasing. *G. lucidum* spores have been reported to exert potential therapeutic and prophylactic effects against these diseases [2,48,49]. The inhibition of key metabolic enzymes, including α-glucosidase, α-amylase, lipase, AChE, and xanthine oxidase, has been confirmed as one of the mechanisms by which *G. lucidum* spore powder confers beneficial effects against these disorders [21,50,51]. In this study, the inhibitory activities against these five enzymes were measured and compared among the nine samples. As shown in Table 8, samples from different regions in China exhibited significant differences in enzyme inhibitory capacities. Notably, sample GZ demonstrated the strongest inhibitory activities against all five enzymes (α-glucosidase, α-amylase, lipase, AChE, and xanthine oxidase), indicating its excellent potential for the prevention and adjunctive management of type II diabetes, obesity, hyperuricemia, and Alzheimer’s disease. Statistical analysis revealed that the significantly lower inhibitory activities (*p* < 0.05) are as follows: against α-glucosidase in samples FJ and LQ; against α-amylase in samples AH and LQ; against lipase in sample LQ; against AChE in samples JL, FJ, and LQ; and against xanthine oxidase in samples DX, FJ, and LQ. Previous studies have reported that the inhibitory effects of *G. lucidum* spore powder on metabolic syndrome-related enzymes are primarily attributed to its core bioactive components, including phenolics, triterpenoids, and polysaccharides [50,51]. Among these components, polysaccharides exhibited inhibitory activity against these enzymes due to the presence of uronic acid and abundant hydroxyl and carboxyl groups in their branched structures [47]. The hydroxyl, methyl, and formyl, and carboxylic acid moieties at specific molecular sites of phenolic compounds and triterpenoids can induce conformational changes in these enzymes, thereby reducing their catalytic activity [52]. Correlation analysis in this work showed that total phenolic content was highly positively correlated with xanthine oxidase and lipase inhibitory activities, with correlation coefficients of 0.833 and 0.767, respectively. Total triterpenoid content was strongly correlated with α-glucosidase and α-amylase inhibitory activities, with correlation coefficients of 0.817 and 0.783, respectively. Polysaccharide content showed a strong positive correlation with α-glucosidase, AChE, and xanthine oxidase inhibitory activities, with correlation coefficients of 0.967, 0.833, and 0.950, respectively (Figure 2).

### 3.9. PCA

**Figure 3:**
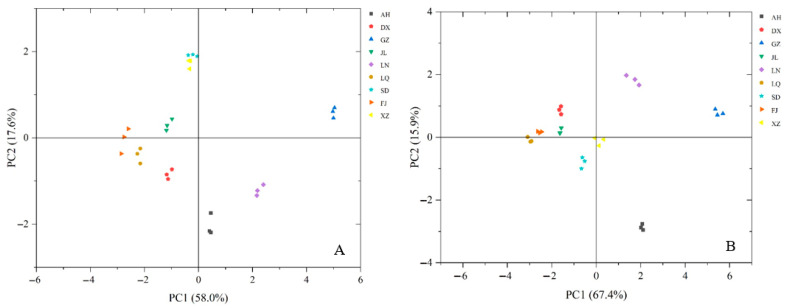
The plot of principal component scores for *G. lucidum* spore powder samples collected from different geographical regions of China. (**A**) Based on nine chemical composition markers. (**B**) Based on ten biological activity indicators. DX, SD, FJ, JL, XZ, LQ, AH, LN, and GZ represent samples collected from Heilongjiang, Shandong, Fujian, Jilin, Xizang, Zhejiang, Anhui, Liaoning, and Guizhou provinces, respectively.

To intuitively visualize the comprehensive quality differences among *G. lucidum* spore powder samples from different geographical origins and to reduce the dimensionality of our high-dimensional multivariate dataset without losing critical information, we performed PCA. Two independent PCA models were constructed: the first was based on nine chemical composition markers (total sugar, reducing sugar, polysaccharide, lipid, protein, total phenolics, total triterpenoids, ergosterol, and total mineral elements) (Figure 3A), and the second was based on ten biological activity indicators (DPPH· scavenging activity, ABTS^+^· scavenging activity, FRAP, CUPRAC, Fe^2+^-chelating capacity, and inhibitory activities against α-glucosidase, α-amylase, lipase, AChE, and xanthine oxidase) (Figure 3B), to systematically differentiate the samples across multiple dimensions.

As shown in Figure 3A, PCA based on chemical composition parameters yielded the first two principal components (PCs), which explained 75.55% of the total variance in the dataset. PC1 was positively correlated with all nine chemical composition variables, while PC2 was positively correlated with the contents of lipids, ergosterol, total triterpenoids, total sugars, and polysaccharides. The nine samples were clearly classified into six groups: SD and XZ, FJ and LQ, and JL and DX were each clustered together, while AH, LN, and GZ each formed an independent cluster.

PCA based on biological activities yielded the first two PCs, which accounted for 83.30% of the total variance in the dataset. PC1 was positively correlated with all ten bioactive indicators, while PC2 was positively correlated with the scavenging activities against DPPH and ABTS radicals, as well as the inhibitory activities against α-glucosidase and α-amylase. As shown in Figure 3B, samples GZ, AH, and LN were clustered with high positive scores on PC1, whereas samples LQ, FJ, DX, and JL were clustered with negative PC1 scores. These results indicated that sample GZ exhibited the strongest overall antioxidant and metabolic enzyme inhibitory activities, followed by samples AH and LN. It should be noted that all antioxidant and metabolic enzyme inhibitory activities of *G. lucidum* spore powders in this study were assessed in vitro. Therefore, the in vivo physiological relevance of these findings requires further verification through animal experiments and clinical trials in future research.

## 4. Conclusions

In summary, our data demonstrate that sporoderm-broken *G. lucidum* spore powder is rich in various bioactive components, including polysaccharides, triterpenoids, phenolics, and ergosterol, and consequently exhibits significant antioxidant and metabolic enzyme inhibitory activities. All tested quality parameters varied significantly among the nine samples collected from representative regions across China. PCA results further confirmed that samples GZ, AH, and LN possess superior comprehensive quality compared to the other samples. It should be noted that the observed quality variations are not solely dependent on geographical origin but are also affected by other factors, including strain characteristics, cultivation methods, processing technologies, and storage duration. Nevertheless, the data obtained in this study provide a comprehensive comparative profile of *G. lucidum* spore powders from different regions in China and serve as a scientific basis for quality control and functional applications.

## Figures and Tables

**Table 1:** The sporoderm-broken rate and color of *G. lucidum* spore powder samples collected from different regions of China.

Sample	Sporoderm-Broken Rate	L*	a*	b*
JL	96.98 ± 1.35% a	18.42 ± 0.10 c	10.25 ± 0.33 c	10.56 ± 0.22 d
AH	99.17 ± 1.44% a	19.09 ± 0.54 b	10.94 ± 0.31 b	11.72 ± 0.24 c
SD	97.82 ± 2.28% a	17.93 ± 0.23 d	9.05 ± 0.02 d	8.14 ± 0.29 e
DX	96.27 ± 3.55% a	17.36 ± 0.35 d	7.71 ± 0.38 e	7.99 ± 0.42 e
GZ	99.98 ± 0.03% a	20.88 ± 0.21 a	11.00 ± 0.26 b	12.55 ± 0.37 b
LN	99.97 ± 0.04% a	14.48 ± 0.35 e	5.69 ± 0.23 f	4.15 ± 0.30 g
FJ	94.08 ± 2.55% a	20.81 ± 0.22 a	11.92 ± 0.18 a	14.15 ± 0.06 a
XZ	96.60 ± 1.42% a	17.75 ± 0.06 d	7.69 ± 0.19 e	6.46 ± 0.16 f
LQ	94.92 ± 4.50% a	20.63 ± 0.20 a	10.29 ± 0.19 c	7.61 ± 0.29 e

Note: JL, AH, SD, DX, GZ, LN, FJ, XZ, and LQ represent samples collected from the provinces of Jilin, Anhui, Shandong, Heilongjiang, Guizhou, Liaoning, Fujian, Xizang, and Zhejiang, respectively. Different letters within the same column indicate significant differences at *p* < 0.05.

**Table 2:** The contents of total sugar, reducing sugar, lipid, and protein in *G. lucidum* spore powder samples collected from different regions of China.

Sample	Total Sugar (mg/g)	Reducing Sugar (mg/g)	Lipid (g/100 g)	Protein (g/100 g)
JL	24.16 ± 0.41 c	2.89 ± 0.09 h	32.10 ± 0.97 cd	7.15 ± 0.78 d
AH	20.96 ± 0.27 d	5.80 ± 0.24 c	31.99 ± 0.92 cd	10.66 ± 0.51 b
SD	23.67 ± 1.19 c	3.20 ± 0.07 g	36.58 ± 0.26 ab	5.34 ± 0.75 e
DX	20.93 ± 0.92 d	4.20 ± 0.08 f	34.85 ± 0.57 b	9.45 ± 0.48 c
GZ	36.02 ± 0.23 a	7.06 ± 0.03 b	37.94 ± 0.68 a	15.35 ± 0.23 a
LN	27.88 ± 0.56 b	9.16 ± 0.01 a	35.34 ± 0.70 b	14.52 ± 0.12 a
FJ	13.55 ± 0.57 f	5.71 ± 0.07 c	32.48 ± 1.32 c	1.96 ± 0.59 f
XZ	21.29 ± 0.58 d	5.54 ± 0.05 d	36.48 ± 0.33 ab	5.50 ± 0.53 e
LQ	18.85 ± 0.49 e	5.13 ± 0.05 e	30.65 ± 0.56 d	10.97 ± 0.52 b

Note: JL, AH, SD, DX, GZ, LN, FJ, XZ, and LQ represent samples collected from the provinces of Jilin, Anhui, Shandong, Heilongjiang, Guizhou, Liaoning, Fujian, Xizang, and Zhejiang, respectively. Different letters within the same column indicate significant differences at *p* < 0.05.

**Table 3:** The contents of polysaccharide, ergosterol, total triterpenoid, and total phenolics in *G. lucidum* spore powder samples collected from different regions of China.

Sample	Polysaccharide (mg/g)	Ergosterol (mg/g)	Total Triterpenoid (mg/g)	Total Phenolics (μg/g)
JL	11.35 ± 0.19 f	1.90 ± 0.22 d	8.44 ± 0.08 d	228.18 ± 2.82 d
AH	14.46 ± 0.13 d	1.03 ± 0.02 e	8.56 ± 0.03 d	293.17 ± 3.49 b
SD	16.06 ± 0.13 c	2.97 ± 0.02 b	8.49 ± 0.11 d	214.87 ± 1.44 e
DX	10.22 ± 0.17 g	0.30 ± 0.06 f	8.61 ± 0.13 d	183.8 ± 3.7 f
GZ	27.47 ± 0.12 a	2.60 ± 0.02 c	14.73 ± 0.5 a	351.07 ± 3.68 a
LN	17.19 ± 0.34 b	1.85 ± 0.05 d	10.29 ± 0.08 b	239.91 ± 4.72 c
FJ	7.28 ± 0.25 h	1.77 ± 0.15 d	7.73 ± 0.11 e	106.69 ± 2.89 i
XZ	11.96 ± 0.4 e	3.41 ± 0.04 a	9.26 ± 0.17 c	168.98 ± 4.46 g
LQ	7.63 ± 0.15 h	1.78 ± 0.07 d	8.26 ± 0.13 d	120.23 ± 2.14 h

Note: JL, AH, SD, DX, GZ, LN, FJ, XZ, and LQ represent samples collected from the provinces of Jilin, Anhui, Shandong, Heilongjiang, Guizhou, Liaoning, Fujian, Xizang, and Zhejiang, respectively. Different letters within the same column indicate significant differences at *p* < 0.05.

**Table 4:** The individual phenolics contents of *G. lucidum* spore powder samples collected from different regions of China (μg/g).

Sample	Protocatechuic Acid	Hydroxybenzoic Acid	Vanillic Acid	Kaempferol	Gallic Acid
JL	40.61 ± 0.95 b	4.97 ± 0.41 d	ND	20.54 ± 0.22 b	2.1 ± 0.18 c
AH	21.19 ± 1.09 e	4.85 ± 0.01 d	ND	18.63 ± 0.70 bc	ND
SD	13.14 ± 0.16 f	4.46 ± 0.05 de	7.05 ± 0.06 b	ND	ND
DX	26.16 ± 0.29 d	7.85 ± 0.70 b	ND	17.77 ± 3.43 bc	1.66 ± 0.26 c
GZ	50.87 ± 1.44 a	9.97 ± 0.34 a	7.05 ± 0.03 b	26.51 ± 0.57 a	10.3 ± 0.78 a
LN	11.94 ± 0.31 f	3.67 ± 0.31 f	4.84 ± 0.05 c	16.62 ± 0.59 c	3.99 ± 0.66 b
FJ	30.27 ± 2.42 c	6.89 ± 0.07 c	ND	10.71 ± 0.97 d	2.27 ± 0.64 c
XZ	7.48 ± 0.28 g	ND	7.88 ± 0.02 a	18.33 ± 0.89 bc	ND
LQ	22.18 ± 0.85 e	4.1 ± 0.33 ef	ND	ND	0.71 ± 0.11 d

Note: JL, AH, SD, DX, GZ, LN, FJ, XZ, and LQ represent samples collected from Jilin, Anhui, Shandong, Heilongjiang, Guizhou, Liaoning, Fujian, Xizang, and Zhejiang provinces, respectively. Different letters within the same column indicate significant differences at *p* < 0.05. ND, not detected.

**Table 5:** The nucleosides contents of *G. lucidum* spore powder samples collected from different regions of China (μg/g).

Sample	Uracil	Adenosine	Uridine	Guanosine	Inosine	Guanine
JL	3.51 ± 0.04 d	36.86 ± 0.17 d	46.87 ± 0.49 e	12.61 ± 0.18 d	ND	10.38 ± 0.23 d
AH	12.66 ± 0.46 b	68.29 ± 1.28 b	96.69 ± 1.13 c	31.41 ± 0.36 b	ND	4.59 ± 0.24 e
SD	3.84 ± 0.04 d	31.44 ± 0.61 e	23.65 ± 0.05 g	ND	ND	100.02 ± 0.68 a
DX	3.87 ± 0.17 d	39.09 ± 0.81 d	39.8 ± 1.01 f	10.11 ± 0.17 f	ND	5.92 ± 0.49 e
GZ	6.54 ± 0.05 cd	64.65 ± 1.30 c	77.67 ± 1.88 d	29.57 ± 0.19 c	11.96 ± 0.10 c	ND
LN	51.98 ± 3.99 a	144.53 ± 2.03 a	210.41 ± 0.41 a	36.56 ± 0.70 a	10.76 ± 0.07 d	66.44 ± 2.01 b
XZ	7.77 ± 0.06 c	38.93 ± 1.30 d	39.32 ± 0.92 f	12.84 ± 0.58 d	18.33 ± 0.77 a	50.38 ± 1.51 c
FJ	7.21 ± 0.04 c	21.71 ± 0.55 f	95.65 ± 0.78 c	10.66 ± 0.02 f	14.74 ± 0.33 b	ND
LQ	6.05 ± 0.00 cd	11.39 ± 0.59 g	102.47 ± 1.30 b	11.40 ± 0.05 e	8.38 ± 0.14 e	ND

Note: JL, AH, SD, DX, GZ, LN, FJ, XZ, and LQ represent samples collected from Jilin, Anhui, Shandong, Heilongjiang, Guizhou, Liaoning, Fujian, Xizang, and Zhejiang provinces, respectively. Different letters within the same column indicate significant differences at *p* < 0.05. ND, not detected.

**Table 6:** The mineral elements of *G. lucidum* spore powder samples collected from different regions of China (mg/kg).

Element	Samples								
	JL	AH	SD	DX	GZ	LN	FJ	XZ	LQ
B	1.31 ± 0.20 f	2.59 ± 0.08 d	2.89 ± 0.08 c	3.13 ± 0.07 c	8.11 ± 0.30 a	1.49 ± 0.13 f	5.23 ± 0.20 b	2.06 ± 0.05 e	0.58 ± 0.06 g
Mg	61.39 ± 6.58 f	311.07 ± 3.23 c	371.68 ± 5.95 b	75.87 ± 0.17 e	196.20 ± 5.30 d	417.61 ± 8.63 a	72.87 ± 2.90 e	366.03 ± 3.40 b	9.47 ± 0.57 g
K	141 ± 1.50 e	315.59 ± 5.07 a	204.92 ± 0.45 d	111.26 ± 2.63 f	253.63 ± 1.15 b	201.60 ± 2.90 d	144.64 ± 3.42 e	227.18 ± 1.19 c	100.71 ± 0.94 g
Ca	240.08 ± 3.18 d	648.89 ± 42.45 a	27.39 ± 14.48 e	324.57 ± 9.71 c	394.56 ± 18.49 b	412.26 ± 47.90 b	408.25 ± 20.21 b	72.24 ± 21.16 e	15.86 ± 8.00 e
Fe	44.71 ± 0.72 a	23.97 ± 7.16 b	3.24 ± 0.08 e	8.85 ± 1.91 cd	47.39 ± 0.93 a	28.11 ± 0.52 b	24.05 ± 0.34 b	4.76 ± 0.74 de	12.14 ± 0.08 c
Se	0.10 ± 0.03 c	0.09 ± 0.01 c	0.45 ± 0.01 a	0.07 ± 0.02 c	0.10 ± 0.01 c	0.50 ± 0.09 a	0.06 ± 0.01 c	0.34 ± 0.06 b	0.08 ± 0.02 c
Mn	3.15 ± 0.02 g	5.79 ± 0.17 a	4.36 ± 0.02 d	3.49 ± 0.07 f	5.07 ± 0.08 b	4.79 ± 0.08 c	2.76 ± 0.03 h	3.79 ± 0.04 e	2.04 ± 0.03 i
Ba	6.71 ± 0.02 c	3.15 ± 0.07 f	1.03 ± 0.02 h	7.99 ± 0.05 b	4.91 ± 0.05 e	2.19 ± 0.01 g	5.21 ± 0.03 d	0.94 ± 0.01 i	8.42 ± 0.03 a
Cu	16.75 ± 0.06 e	13.49 ± 0.32 g	20.39 ± 0.14 a	16.53 ± 0.09 e	15.39 ± 0.03 f	19.94 ± 0.13 b	17.31 ± 0.07 d	19.46 ± 0.17 c	17.26 ± 0.04 d
As	0.09 ± 0.001 e	0.06 ± 0.002 f	0.05 ± 0.001 f	0.12 ± 0.002 d	0.19 ± 0.01 b	0.16 ± 0.01 c	0.20 ± 0.01 a	0.06 ± 0.004 f	0.05 ± 0.002 f
Ti	0.96 ± 0.03 e	1.42 ± 0.11 b	1.06 ± 0.03 d	1.46 ± 0.03 b	1.10 ± 0.02 d	1.89 ± 0.003 a	0.74 ± 0.004 f	1.33 ± 0.03 c	0.31 ± 0.02 g
Cd	0.17 ± 0.003 c	0.23 ± 0.003 a	0.05 ± 0.001 g	0.16 ± 0.001 d	0.17 ± 0.002 c	0.08 ± 0.001 e	0.17 ± 0.003 c	0.07 ± 0.001 f	0.21 ± 0.001 b
Cr	0.12 ± 0.01 f	1.25 ± 0.05 a	0.48 ± 0.01 c	0.32 ± 0.02 e	0.87 ± 0.01 b	0.50 ± 0.01 c	0.37 ± 0.01 d	0.34 ± 0.02 de	0.13 ± 0.004 f
Pb	0.06 ± 0.001 e	0.07 ± 0.002 d	0.18 ± 0.001 c	0.20 ± 0.003 b	0.05 ± 0.002 f	0.52 ± 0.01 a	0.05 ± 0.002 f	0.20 ± 0.002 b	0.03 ± 0.004 g
Total	516.61 ± 8.88 e	1327.65 ± 64.54 a	638.19 ± 17.57 d	554.04 ± 10.5 e	927.74 ± 16.31 c	1091.65 ± 42.99 b	681.95 ± 25.87 d	698.8 ± 24.13 d	167.31 ± 8.05 f

Note: JL, AH, SD, DX, GZ, LN, FJ, XZ, and LQ represent samples collected from Jilin, Anhui, Shandong, Heilongjiang, Guizhou, Liaoning, Fujian, Xizang, and Zhejiang provinces, respectively. Different letters within the same row indicate significant differences at *p* < 0.05.

**Table 7:** The antioxidant capacities of *G. lucidum* spore powder samples collected from different regions of China.

Sample	DPPH· Scavenging (mg VCE/g)	ABTS^+^· Scavenging (mg VCE/g)	FRAP (mg VCE/g)	CUPRAC (mg VCE/g)	Fe^2+^-Chelation (mg EDTA/g)
JL	0.31 ± 0.004 e	0.87 ± 0.05 f	0.30 ± 0.001 f	2.67 ± 0.09 d	0.25 ± 0.02 de
AH	0.34 ± 0.007 c	0.97 ± 0.02 c	0.42 ± 0.001 a	3.70 ± 0.03 b	0.72 ± 0.02 a
SD	0.26 ± 0.01 g	0.95 ± 0.05 cd	0.31 ± 0.01 e	2.64 ± 0.09 d	0.29 ± 0.01 cd
DX	0.34 ± 0.003 c	0.84 ± 0.01 f	0.36 ± 0.001 c	1.59 ± 0.04 f	0.25 ± 0.01 de
GZ	0.40 ± 0.002 a	1.46 ± 0.003 a	0.42 ± 0.003 a	4.02 ± 0.07 a	0.52 ± 0.02 b
LN	0.39 ± 0.002 a	1.40 ± 0.03 b	0.34 ± 0.001 d	3.18 ± 0.04 c	0.33 ± 0.06 c
FJ	0.32 ± 0.003 d	0.76 ± 0.01 g	0.31 ± 0.001 ef	1.99 ± 0.03 e	0.23 ± 0.02 de
XZ	0.36 ± 0.004 b	0.90 ± 0.02 de	0.41 ± 0.004 b	2.71 ± 0.10 d	0.28 ± 0.04 cd
LQ	0.29 ± 0.001 f	0.92 ± 0.02 cde	0.31 ± 0.007 ef	1.55 ± 0.01 f	0.21 ± 0.03 e

Note: JL, AH, SD, DX, GZ, LN, FJ, XZ, and LQ represent samples collected from Jilin, Anhui, Shandong, Heilongjiang, Guizhou, Liaoning, Fujian, Xizang, and Zhejiang provinces, respectively. Different letters within the same column indicate significant differences at *p* < 0.05.

**Table 8:** The enzyme inhibitory activities of *G. lucidum* spore powder samples collected from different regions of China.

Sample	α-Glucosidase Inhibition (mg ACE/g)	α-Amylase Inhibition (mg ACE/g)	Lipase Inhibition (μg ORE/g)	AChE Inhibition (μg GTE/g)	Xanthine Oxidase Inhibition (μg APE/g)
JL	0.49 ± 0.03 ef	5.55 ± 0.41 d	0.95 ± 0.01 d	5.43 ± 0.46 e	1.52 ± 0.05 e
AH	0.56 ± 0.06 de	0.94 ± 0.03 g	1.11 ± 0.01 b	17.64 ± 0.69 b	5.57 ± 0.39 c
SD	0.81 ± 0.03 c	2.52 ± 0.32 f	0.93 ± 0.01 e	14.73 ± 0.50 c	6.71 ± 0.44 b
DX	0.43 ± 0.02 fg	8.18 ± 0.58 c	0.85 ± 0.005 g	8.64 ± 0.57 d	0.92 ± 0.03 f
GZ	1.97 ± 0.12 a	12.16 ± 0.41 a	1.21 ± 0.012 a	22.99 ± 0.74 a	9.85 ± 0.62 a
LN	1.15 ± 0.05 b	9.48 ± 0.33 b	0.88 ± 0.011 f	15.30 ± 2.18 c	4.94 ± 0.25 d
FJ	0.35 ± 0.01 g	3.61 ± 0.2 e	0.84 ± 0.003 g	5.90 ± 0.37 e	0.59 ± 0.05 f
XZ	0.62 ± 0.05 d	5.62 ± 0.34 d	0.98 ± 0.004 c	13.31 ± 1.45 c	1.61 ± 0.05 e
LQ	0.33 ± 0.02 g	0.8 ± 0.02 g	0.82 ± 0.002 h	5.51 ± 0.23 e	0.84 ± 0.07 f

Note: JL, AH, SD, DX, GZ, LN, FJ, XZ, and LQ represent samples collected from Jilin, Anhui, Shandong, Heilongjiang, Guizhou, Liaoning, Fujian, Xizang, and Zhejiang provinces, respectively. Different letters within the same column indicate significant differences at *p* < 0.05.

## Data Availability

The original contributions presented in this study are included in the article/Supplementary Materials. Further inquiries can be directed to the corresponding authors.

## Supplementary Materials

The following supporting information can be downloaded at: https://www.mdpi.com/article/10.3390/foods15091579/s1, Table S1: Experiment Data.
